# 

**Published:** 1851-01

**Authors:** 


### CONTENTS

### OF NO. I.

---

### ORIGINAL COMMUNICATIONS.

### ESSAYS AND CASES.

ART. I.—Clinical Observations in Private Practice. By Dr. E. B. HASKINS, of Clarksville, Tenn. - - - 1

ART. II.—Catalogue of inorganic substances employed in medicine, with the powers of each substance. Prepared from the manuscripts of Dr. WM. TULLY, with a system of powers in the order and kind of their operation. - 11

ART. III.—Continuation of the "Case of Facial Palsy consequent upon Otitis," reported for the December number, 1849, of this Journal. By W. H. COBB, M. D., of Louisville, Ky. - - - - - - - - - 19

ART. IV.—Small Pox—Vaccination and Revaccination—their present position. By THEODORE S. BELL, M. D., of Louisville, Ky. - - - - - - - - - 22

### BIBLIOGRAPHICAL NOTICES.

ART. V.—Woman: her diseases and remedies. A series of letters to his class, by CHARLES D. MEIGS, M. D., Professor of Midwifery, and the diseases of women and children, in the Jefferson Medical College at Philadelphia, &c. &c. - - - - - - - - - 77

ART. VI.—Renal Affections: their diagnosis and pathology. By CHARLES FRICK, M. D. - - - - - - - 77

Art. VII.—The Races of Men: A Fragment. By Robert Knox, M. D. - - - - - - - - 79

Art. VIII.—Silliman's Journal. - - - - - - 80

SUMMARY OF RECENT MEDICAL INTELLIGENCE.

Dengue Fever, - - - - - - - - - - 82
Surgical Treatment of Epilepsy, - - - - - - - 85
On the Action of Mercurial Ointment and the Vapor of Mercury, 87
The Odor of Musk destroyed by Camphor, - - - - - 88
On Cod-Liver Oil in Phthisis, - - - - - - - - 88
On Linseed Oil in Hemorrhoids, - - - - - - - 88
On Syrupus Iodidi Ferri, - - - - - - - - - 89
On the Acetate of Potash in some Diseases of the Skin, - - 90
Water-Mellon Seed as a Dieuretic. - - - - - 91
Professor Eve's Card, - - - - - - - - 92

